# Is a 4 J/cm^2^ PpIX-Weighted Simulated Daylight (SDL-PDT) Dose Still Efficient for Photodynamic Therapy of Actinic Keratosis?

**DOI:** 10.3390/ph16101454

**Published:** 2023-10-13

**Authors:** Mathilde Fronville, Muriel Creusot, Serge R. Mordon

**Affiliations:** 1 Centre Dermatologique du Roy, Plancenoit, 1380 Lasne, Belgium; mathilde.fronville@gmail.com (M.F.); muriel.creusot@gmail.com (M.C.); 2 Hemerion Therapeutics, 59650 Villeneuve d’Ascq, France

**Keywords:** actinic keratosis, photodynamic therapy, protoporphyrin IX, artificial daylight, indoor daylight, simulated daylight, white light, 5-ALA, clinical study

## Abstract

**Background:** Several solutions are now proposed to provide indoor illumination with so-called **artificial** white **light** or simulated **daylight** (SDL-**PDT**), resulting in an effective treatment for actinic keratosis (AK). However, the optimal PpIX-weighted light dose is still debated. Integrating the effective irradiance over the irradiation time yields the effective light dose, which is also known as the protoporphyrin IX-weighted light dose and is a key parameter for the efficacy of the treatment. **Objectives:** The paper aims to report the clinical outcomes of SDL-PDT when using the PpIX-weighted light dose of 4 J/cm^2^, in patients treated for AK lesions of the scalp or the face at our medical dermatology center (ClinicalTrials.gov NCT052036). **Methods:** A total of 30 patients (16 males, 14 females), with a mean age of 71.0 ± 10.2, with phototype 1 (16 patients) and phototype 2 (14 patients) with grade I–II AK were treated with a drug light interval (DLI) of 10 min and a light exposure of 35 min (Dermaris, Surgiris, Croix, France), corresponding to a PpIX-weighted light dose of 4 J/cm^2^. The primary endpoint was the cure rate of patients at six months post-treatment. Secondary endpoints included scores of pain, erythema, crusts, and discomfort during or/and post the treatment. **Results:** In total, 762 AK were treated. Six months following treatment, the cure rate of the patients was 77%. The median pain score was less than 1 out of 10 for most of the patients. Erythema was observed in all patients and lasted 3 days (±1.5 day). Crusts were seen in 28 patients. Discomfort was reported as mild or less in more than 97% of patients. **Conclusions:** The shortening of the PpIX-weighted light dose to 4 J/cm^2^, corresponding to an illumination duration of 35 min with the Dermaris, does not modify the efficacy of the SDL-PDT. This observation is in agreement with recent published data demonstrating that the light dose can be reduced. Furthermore, this clinical study confirmed that SDL-PDT is an effective and nearly painless treatment with minimal side effects for patients with AK lesions of the scalp.

## 1. Introduction

Actinic keratosis (AK) is a chronic skin disease with a high prevalence in the Caucasian population that has increased to even higher numbers in recent years. The prevalence is estimated to be 20–30% in Europe and 40–50% in Australia, in individuals older than 40 years of age. AK lesions are located on sun-exposed skin and are more frequent in older patients with fair skin. [1]. If the greatest degree of improvement is seen with photodynamic therapy (PDT), other modalities such as cryotherapy, imiquimod, ingenol mebutate (IMB), 5-fluorouracil (5-FU), trichloroacetic acid (TCA), and ablative fractional laser (AFXL) can also be used [2].

The development of visible dysplastic lesions (actinic keratosis—AK) and subsequent progression to invasive SCC (squamous cell carcinoma) requires an effective treatment. It is important to not only treat the AK itself, but also the area of photo-damaged skin around the AK with subclinical (neither visible nor palpable) lesions known as field cancerization [3]. Topical photodynamic therapy (PDT) has recently become a good treatment option for actinic keratosis and basal cell carcinoma, especially when treating large areas and areas with field cancerization [4].

For AK treatment, PDT requires topically applied precursors like 5- or delta-aminolevulinic acid (ALA) or its methyl ester the methyl-aminolevulinate (MAL). The 5-ALA is not a photosensitizer but a biological precursor. The 5-ALA is converted to the photosensitizing protoporphyrin IX (PpIX) within the mitochondria in the heme biosynthetic pathway. The observed overall increase in the ability of protoporphyrin IX (PpIX) synthesis by dysplastic cells compared to that of normal cells has been related to the increased activity of most enzymes involved in heme biosynthesis and decreased activity of ferrochelatase [5].

In order to perform the illumination, PDT using daylight as the photoactivating light source (DL-PDT) has been proposed as an attractive treatment option for actinic keratosis (AK). DL-PDT has proven to be similarly effective as conventional PDT (C-PDT) while being nearly painless [6,7]. DL-PDT is particularly relevant for patients with large areas of field cancerization, as the size of the treatment area has been reported to be the strongest predictor of pain [8]. Unfortunately, DL-PDT requires weather conditions and temperatures to be suitable for effective treatment and for patients to stay comfortably outdoors for two hours or more [9]. Moreover, the light dose delivered during DL-PDT varies considerably depending on the weather conditions, the location, the time of the year, and the day time [9].

For example, Cordey et al. reported their experience of daylight photodynamic therapy in 64 patient treatment courses. They showed that most patients (73%) achieved clearance or at least a good response to treatment and had high levels of satisfaction with the daylight photodynamic therapy. However, daylight exposure measurements indicated that treatment is feasible in Scotland between April to September [10]. Moreover, production of PpIX was found to be a strongly temperature-dependent process. Consequently, temperature may be a limiting factor, however, as studies in vitro and in mice suggest, low temperatures reduce PpIX production. Low temperatures also make it difficult for the patient to stay outside during exposure. A temperature range of 10 °C to 35 °C for daylight PDT has been recommended [11,12].

Due to the limitations of DL-PDT, artificial daylight sources aimed at allowing simulated daylight PDT (SDL-PDT), also referred to as artificial or indoor daylight PDT, are emerging for the treatment of AK. Contrary to DL-PDT, SDL-PDT can be conducted in all weather conditions, in any geographic location, year-round, and always delivers the same light dose. A non-exhaustive review of commercially available light sources designed or proposed for SDL-PDT of AK has been published [13].

Recent studies have clearly demonstrated that SDL-PDT is an efficient option to treat AK [14]. Maire et al. obtained excellent results with SDL-PDT with a 90% clearance rate. However, the main limitation of this treatment technique was the duration of the light exposure (typically 150 min) which was much longer than the conventional PDT with C-PDT (typically 9 min) even if a long incubation time (2 to 3 h) is required before illumination. More recently, Creusot and Mordon, when using the Dermaris, demonstrated that using only one hour of low-intensity light exposure could lead to a 93% cure rate [15]. In that case, the PpIX-weighted daylight was only 8 J/cm^2^. Similarly, Arisi et al. performed a randomized split-face clinical trial of conventional vs. indoor daylight photodynamic therapy for the treatment of multiple actinic keratosis of the face and scalp. Using the Dermaris device for SDL-PDT, they showed that SDL-PDT may represent an alternative treatment protocol to C-PDT for the in-office treatment of AKs patients with better tolerability and without inferior efficacy. A total of 70% of the patients gave their overall preference for SDL-PDT [16].

However, the light doses needed to achieve the effective treatment of AKs vary between the studies of daylight-PDT, and the association between light dose and efficacy is, therefore, still unclear. Several authors have previously determined the minimal total PpIX-weighted daylight dose for full efficacy to be 4–8 J/cm^2^ [17,18,19,20,21]. Consequently, this study aims to assess the efficacy and tolerability of SDL-PDT using the Dermaris and to confirm these previous findings using the lowest dose of 4 J/cm^2^ determined by these authors.

## 2. Results 

### 2.1. Population Study

Thirty patients (21 males, 9 females) were included in the study and were evaluated at 3 months and 6 months following the initial treatment. Demographics and clinical characteristics are given in Table 1, including mean age (71.6 ± 10.3), phototype 1 (16 patients), and phototype 2 (14 patients) with grade I to II AK of the scalp. A total of 762 lesions were treated: grade I (561) and grade II (201).

### 2.2. Efficacy

At the 3 month follow up, the clearance rate of AKs was 53%. All 30 patients had a second session at 3 months to clear remaining grade I and grade II lesions. At the six month follow up, the clearance rate of AKs six months after the second treatment was 77%.

### 2.3. Tolerability

#### 2.3.1. Pain

Twenty-six patients (87%) experienced no pain during the first PDT treatment, while pain was scored as 1 by the remaining four patients (13%). With pain scores ranging from 0 to 1, SDL-PDT using the Dermaris was almost painless.

#### 2.3.2. Crusts

At seven days, crusts using the 6-point scale were scored 0 for 4 patients, 1 for 1 patient, 2 for 9 patients, 3 for 8 patients, 4 for 4 patients, and 5 for 4 patients. For all patients, crusts were healing at seven days after the treatment and were expected to be completely gone three days later according to the dermatologist.

#### 2.3.3. Erythema

Erythema was observed in all 30 patients and lasted 3 days (±1.5 day).

#### 2.3.4. Patient Discomfort

Discomfort was scored as mild (63%) or none (34%) for 29 patients. Only one patient experienced moderate discomfort during the treatment. Quite similar results were obtained regarding discomfort during the six days post-treatment. Consequently, no discomfort rated as more than moderate was reported at any time of the study.

## 3. Discussion

The clearance rates observed at the 3 month follow up (52%) and the 6 month follow up (77%) in this clinical study using a 4 J/cm^2^ PpIX-weighted simulated daylight (SDL-PDT) dose were similar or even higher than those reported in previous studies. As regards, MAL-PDT data obtained were 48% for AK I and 69.8% for AK II [22]. Similarly, Salido-Valejo observed a cure rate of 56.8% in the patients treated with DL-PDT. [23]. However, a better evaluation of the reduction in AK and inflammation severity could be obtained by using devices such as the Antera machine [24].

The light doses needed to achieve effective treatment of AKs vary between the studies of daylight-PDT, and the association between light dose and efficacy is, therefore, still unclear.

Wiegell et al. stated in 2009, when using DL-PDT, that more than 4 to 8 J/cm^2^ PpIX-weighted daylight is required for the maximum cure of AK [17].

In 2012, in a study comparing 90 min and 150 min of natural daylight illumination (DL-PDT) performed by Wiegell et al., no association was found between the response rate and light dose in patients who received a protoporphyrin IX-weighted dose greater than 3.5 J.cm^2^ [25]

Previous studies have clearly demonstrated that SDL-PDT using the Dermaris (as administered in this study) is an effective and nearly painless treatment for patients with AK lesions of the scalp [26,27]. However, until now, the main limitation of this treatment is the duration of the light exposure (typically 150 min) which is much longer than the conventional PDT with red light (typically 9 min) even if a long incubation time (2 to 3 h) is required before illumination. Recently, Creusot and Mordon showed that good efficacy (93% cure rate) and excellent tolerability of SDL-PDT could be obtained with the Dermaris and an effective protoporphyrin IX-weighted dose of 8 J/cm^2^ due to the reduction in the light exposure duration to 1 h [15]. The actual study clearly demonstrated that the protoporphyrin IX-weighted dose can be reduced to 4 J/cm^2^, confirming previous observations by Wiegell and his group.

This observation is also confirmed by a recent study published by Ruiz et al. In their study, they determined the PpIX-effective fluence at varying depths necessary to provide adequate comparisons of the delivered dose from PDT light sources. To perform, their calculations, they measured the irradiance spectrum of each light source using a spectroradiometer in order to obtain calibrated spectral irradiance values in the 380–780 nm range. The measured spectral irradiance was multiplied by the normalized PpIX absorption to calculate the PpIX-effective spectral irradiance. These calculations were performed for four sources used in the literature for the PDT treatment of AKs by PDT: (i) outdoor daylight, (ii) indoor daylight, (iii) RhodoLED lamp (red light), and (iv) BLU-U lamp (blue light). They concluded that the broad spectrum of the daylight sources resulted in an effective spectrum containing wavelengths for the Soret and Q-bands of PpIX absorption. The outdoor daylight effective irradiance was ~2X that of the indoor daylight due to the window transmission attenuation of the UV/blue wavelengths. However, the presence of UV must be avoided during outdoor illumination. Consequently, the application of a chemical sunscreen to the treatment site and entire sun-exposed area is compulsory before sending the patient outside for the PDT session [28].

Finally, the results also showed that despite the >2× differences in irradiance, the RhodoLED lamp and indoor daylight had only ~10% difference in their PpIX-effective intensities [29].

SDL-PDT using the Dermaris, can be easily administered in our medical dermatology center. These treatment parameters differ somewhat from those used in DL-PDT, which commonly involves a drug light interval (DLI) of 30 min. In our study, the DLI was only 10 min. After the application of MAL to the scalp, 10 min was the mean duration required to install the patient in the room and start the illumination with the Dermaris. It is now clearly demonstrated that a short DLI is associated to a low pain score. A recent review has confirmed the advantage of reducing the DLI [30]. When using ALA or MAL, it is important to avoid the confusion between incubation time and DLI. When performing ALA-PDT or MAL-PDT, DLI is the period of time between first PpIX production by dysplastic cell and its activation by light. Studies of intracellular PpIX formation kinetics have demonstrated that PpIX formation by dysplastic cells is quasi-instantaneous after 5-ALA administration. Since ALA or MAL is not removed, formation of PpIX will continue as long as the dysplastic cell is alive. Short DLI are associated with reduced PpIX buildup in target tissue due to the absence of PpIX diffusion into surrounding tissues containing intact sensory nerve fibers.

In 2008, Wiegell et al. performed a randomized controlled study to compare conventional red light-emitting diode (LED) light vs. daylight. One area was illuminated by red LED light (37 J/cm^2^) after a 3 h incubation with MAL under an occlusive dressing; the other area was treated with daylight for 2.5 h after the MAL cream had been placed under the occlusive dressing for only half an hour. A reduction of 79% in the daylight area of actinic keratosis (AK) lesions and 71% in the LED area were observed. However, daylight was significantly less painful than LED light with a mean maximal pain score during the daylight exposure of 2.0 (SD ±1.9) compared with 6.7 (SD ±2.2) during red LED exposure (*p* < 0.0001) [31].

In the literature, many measures have been reported to reduce pain during C-PDT including topical or injected local anesthetic and cooling by fans or spraying water on the lesion. The few studies evaluating the effect of these treatments have not proved the effectiveness of any of these treatments [32,33,34,35].

Gandy et al. have also proposed a novel protocol to effectively treat AKs with PDT that eliminates the pre-illumination incubation period in order to reduce the pain. The clinical evaluation consisted of a conventional preparation of the lesion by scrubbing the face and scalp with warm soapy water and descaling hypertrophic AKs with a 4 mm non-disposable curette. ALA was then applied to the patient’s face and scalp before placing them under the blue light for 33 min and 20 s (two cycles of 16 min and 40 s). With this protocol, the patient tolerated the complete course of the treatment and reported no pain (0 out of 10). At one week, the treated areas revealed resolving erythema and desquamation, indicating a good response to the therapy [36]. Mordon et al. performed, on 47 patients, a randomized, controlled, multicenter, intraindividual clinical study. One area was illuminated by red LED light (37 J/cm^2^) after 3 h incubation with MAL under an occlusive dressing; the other with a new device for 2.5 h after the MAL cream had been applied for only half an hour. The clearance rate was similar for the two sides (94.2% vs. 94.9%). However, the pain score was significantly lower for the 30 min DLI compared to the 3 h DLI, respectively, 0.3 vs. 7.4 (*p* < 0.001) [37].

Consequently, short DLI should be used since it is a very simple way to reduce pain. Thereby, the reduction in the DLI is the main contributor of the low pain experienced by the patients in this study: twenty-six patients (87%) experienced no pain during the treatment, while pain was scored as 1 by the remaining four patients (13%). With pain scores ranging from 0 to 1, the treatment was almost painless. The PDT treatment induces an inflammatory reaction which is proportional to the number of lesions [38]. At last, discomfort was reported as mild or less in more than 97% of patients, which makes this treatment well accepted by our patients.

Being an effective and nearly painless treatment for patients, SDL-PDT using the Dermaris, as administered in this study, is a convenient and attractive way for health professionals to deliver PDT treatment of the scalp or the face. First, given the simplicity of the treatment, the minor side effects (i.e., crusts, discomfort) and the absence of serious adverse events, only few minutes (five minutes on average) are required to provide treatment information to patients. This can be performed, therefore, by the dermatologists themselves. Second, the start of photoactivation almost immediately after the application of MAL cream (DLI: 10 min) allows for rapid treatment implementation. Third, as clearly demonstrated in this study, the effective light dose can be reduced to 4 J/cm^2^, leading to a reduction in the illumination time to 35 min which makes this technique more attractive for a private practice. Moreover, the absence of treatment discontinuations due to pain and the high patient acceptability of treatment ensures the smooth running of the treatment.

By contrast with DL-PDT, that can be conducted only in suitable temperature and weather conditions [9], SDL-PDT using the Dermaris is usable year-round in all weather conditions and geographical locations. Indeed, for a given photoactivation time, the Dermaris always delivers the same light dose, which is not the case with daylight. This constant light dose guarantees a high level of reproducibility of the treatment effect. Moreover, contrary to daylight, the Dermaris does not emit ultraviolet radiation and does not require the use of a carefully selected absorbent sunscreen, whereas the European guidelines recommend its use for DL-PDT [39].

## 4. Materials and Methods

### 4.1. Study Design

This prospective study aimed to evaluate the efficacy and tolerability of SDL-PDT using the Dermaris with only a protoporphyrin IX-weighted dose of 4 J/cm^2^, corresponding to a 35 min illumination. The study was approved by the Ethical Clinical Committee of the Centre Dermatologique du Roy and conducted in accordance with the ethical principles of the Declaration of Helsinki (2008) and the International Conference on Harmonization—Good Clinical Practices, and in compliance with local regulatory requirements. The study was prospectively registered at clinicaltrials.gov (ClinicalTrials.gov Identifier: NCT05522036) [40].

### 4.2. Study Population

Patients were recruited from the patient population treated for AK lesions of the scalp at our medical dermatology center from January 2022 to June 2023.

Only patients with a minimum of 9 clinically diagnosed grade I and II AK lesions of the scalp (according to the classification by Olsen et al. [41]), suggesting the existence of field cancerization, were included in the study. All patients were informed about the purpose of the study and gave informed consent before inclusion.

### 4.3. Treatment Procedure

**Figure 1:**
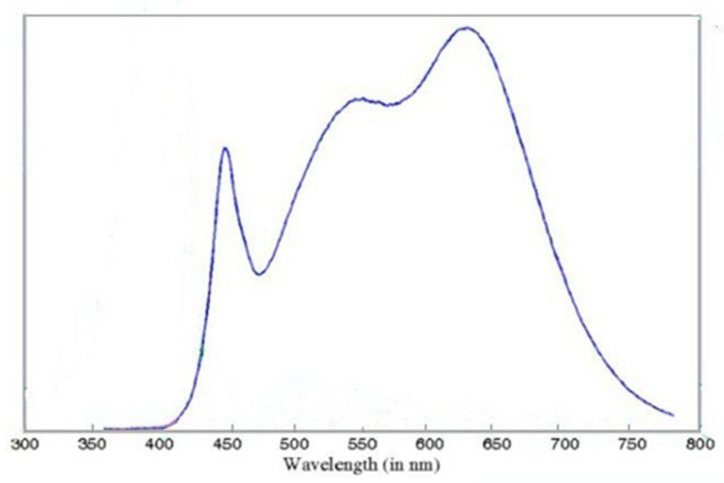
Spectrum of the Dermaris (source Surgiris, Croix, France).

Specifically designed for SDL-PDT and approved for marketing in Europe, the Dermaris (Surgiris, Croix, France) provides uniform photoactivation over a large circular area of approximately 320 cm^2^. Its emission spectrum is given in Figure 1. A gentle sandpaper curettage was performed prior MAL application.

The illumination began 10 min after the application of MAL cream to the treatment area. In this study, the duration of the light exposure was reduced to 35 min to achieve a PpIX-weighted daylight of 4 J/cm^2^. MAL cream was removed after the illumination procedure leading to an incubation time of 45 min.

### 4.4. Endpoints

This clinical study aimed to determine the cure rate of AK lesions at six months after the treatment. Pain during the treatment, discomfort, and erythema were evaluated six days post-treatment, and crusts at seven days after the treatment were also evaluated.

The pain experienced during the first **PDT treatment** was scored on a visual analogue **scale** (VAS) ranging from 0 (no pain) to 10 (worst imaginable pain).

Discomfort during the treatment, which arises from the need for patients to stay under the Dermaris during the 35 min of photoactivation, includes joint pain, fatigue, and refraining from going to the toilet, whereas discomfort at six days post-treatment refers to patients’ quality of life, including difficulty sleeping, inconvenience regarding head care, and embarrassment associated with outings.

The number of AK lesions at the three month and six month follow up visits were clinically evaluated by a dermatologist. The crusts were rated using a 6-point scale (0 stands for no crusts, 1 for very mild crusts, 2 for mild crusts, 3 for moderate crusts, 4 for severe crusts and 5 for very severe crusts). The presence of erythema was noted.

### 4.5. Treatment

Demographic and clinical characteristics of patients were collected at the screening visit. On the day of treatment (within 4 weeks following the screening visit), 2 g of MAL cream (Metvix, Galderma) was applied on lesions and the surrounding normal skin following standard skin preparation (crusts removal and gentle scraping of the lesion surface). After 10 min, the Dermaris was placed 20 cm from the scalp and switched on for 35 min. Specifically designed for SDL-PDT and approved for marketing in Europe, the Dermaris (Surgiris, Croix, France) provides uniform photoactivation over a large circular area of approximately 320 cm^2^. Equipped with white light-emitting diodes, the Dermaris emits a broad spectral range of wavelengths, which overlaps with the protoporphyrin IX (PpIX) absorption spectrum. An irradiance of 2.9 mW/cm^2^ at a distance of 20 cm from the Dermaris is indicated in the technical documentation provided by the manufacturer (https://dermaris-pdt.com/en/dermaris/, accessed on 10 October 2023). Of benefit is the specific design of this light source, as the illumination area is homogenous [15].

Once SDL-PDT using the Dermaris was completed, pain level and discomfort during the treatment were scored by the patient.

A first follow-up visit was scheduled seven days after the treatment. At this visit, crusts were clinically rated by a dermatologist, while patients scored the discomfort they had experienced during the six days post-treatment.

At the second follow-up visit, conducted three months after the treatment, AK lesions were counted by a dermatologist without differentiation between new and recurrent lesions. A second SDL-PDT using the Dermaris was performed on the day if AK lesions were still present. At last, in case of remaining AKs observed at the six month follow up, a third treatment session with the same parameters was carried out.

### 4.6. Data Analysis

Continuous variables are expressed as the means and standard deviations (SD). Analyses were descriptive and were conducted using Microsoft Excel. Excel 2016 (version 16.0).

## Figures and Tables

**Table 1:** Demographics and clinical characteristics.

Age (years)	Total (n = 30)
Mean ± SD	71.6 ± 10.3
Range	54–91
Sex	Number of patients
Male	21 (71%)
Female	9 (29%)
Fitzpatrick skin phototype	Number of patients
1	16 (53%)
2	14 (47%)
AK grade	Number of lesions before PDT
I	561 (74%)
II	201 (26%)
AK grade (% reduction)	Number of lesions after the first PDT session
I	53% (261)
II	45% (110)
AK grade (% reduction)	Number of lesions after the second PDT session
I	74% (145)
II	84% (33)

## Data Availability

Data is contained within the article.
